# Mid-Term to Long-Term Outcomes of Total Hip Arthroplasty Using a Cementless Trochanteric Sparing Short Stem Through Direct Anterior Approach: A Single-Center Study

**DOI:** 10.1016/j.artd.2025.101623

**Published:** 2025-02-07

**Authors:** Seyed Mohammad Javad Mortazavi, Pouya Tabatabaei Irani, Mohammad Poursalehian, Mahsa Mahanrad, Peyman Mirghaderi, Mohammadreza Razzaghof, Sadegh Saberi

**Affiliations:** Joint Reconstruction Research Center, Orthopedic Surgery Department, Imam Khomeini Hospital, Tehran University of Medical Sciences, Tehran, Iran

**Keywords:** Total hip arthroplasty, Survival, Fitmore, Femoral stem, Short stem

## Abstract

**Background:**

Total hip arthroplasty (THA) is increasingly performed in younger patients, necessitating long-term femoral bone preservation. Metaphyseal engaging short stems offer potential benefits by reducing stress shielding and preserving bone stock. However, lacking long-term data in large quantities and younger patients in the literature led this study to assess mid-term to long-term outcome of these short stems.

**Methods:**

This retrospective study evaluated the long-term outcomes of 755 hips (667 patients) underwent THA using the Fitmore stem via a direct anterior approach. Clinical and radiographic assessments were conducted, and survival rates were determined using Kaplan-Meier analyses. Statistical analyses were performed to identify associations and predictors of stem revision.

**Results:**

The overall survival rate for the Fitmore stem was 92.11% at an average follow-up of 10 years. No revisions were performed due to aseptic loosening of the femoral component. Stem revisions were performed in 20 hips, primarily due to periprosthetic fractures followed by periprosthetic joint infections and recurrent dislocations. The clinical outcomes showed significant improvements in HHS, WOMAC Index, and VAS pain scores. Radiographic analysis revealed acceptable rates of complications, with minimal stem subsidence, no severe bone loss, and a low incidence of radiolucent lines and cortical hypertrophy.

**Conclusions:**

The Fitmore stem demonstrated favorable mid-term to long-term outcomes in terms of implant survival, functional scores, and radiographic assessments even in younger populations. The findings contribute to the existing body of knowledge on the Fitmore stem’s efficacy and safety in preserving bone and achieving satisfactory clinical outcomes in THA.

**Level of evidence:**

IV.

## Introduction

**Figure 1:**
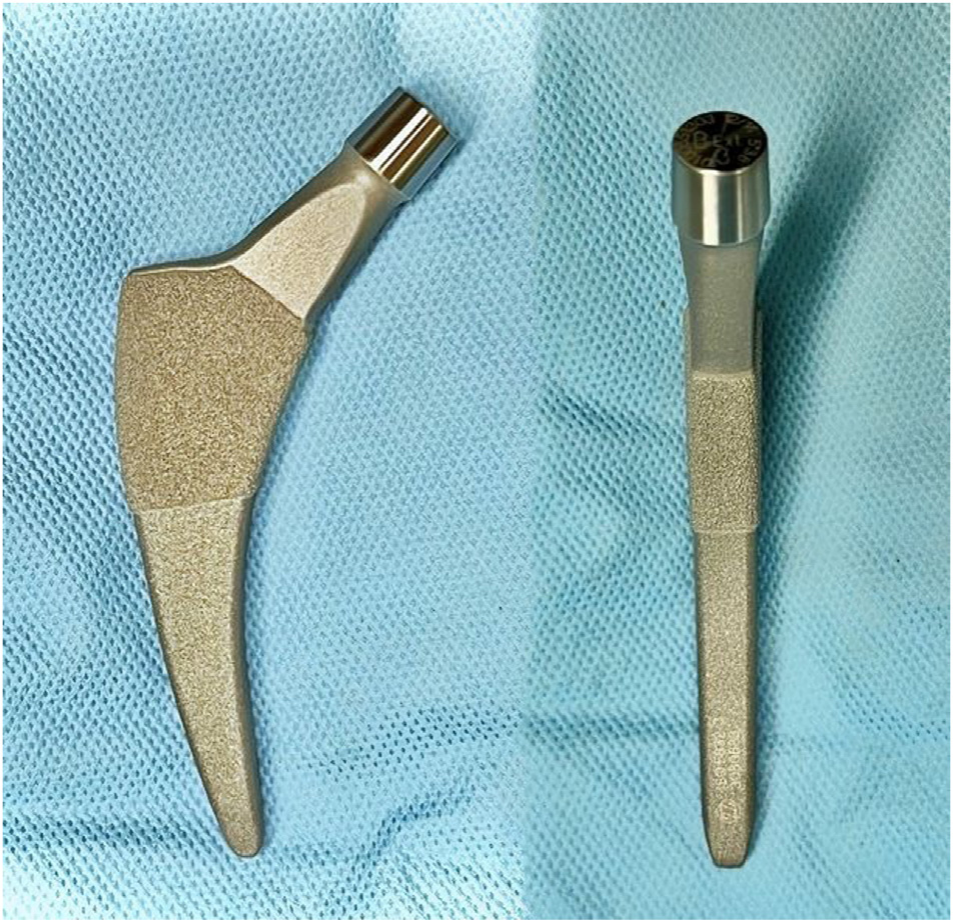
Fitmore stem a short curved cementless trochanteric sparing stem with titanium alloy porous coating.

Recent strides in medical science have contributed to both an extension in life expectancy and an earlier diagnosis of hip joint degenerative diseases. As a result, total hip arthroplasty (THA) is becoming more prevalent, especially among a younger, active demographic [[1], [2], [3], [4]]. This shift underscores the importance of long-term femoral bone preservation. Although conventional stems in THA boast impressive survival rates, issues such as proximal stress shielding, thigh pain, and proximal-distal mismatch remain [5]. To mitigate these challenges, the use of metaphyseal engaging short stems has been suggested [[6], [7], [8], [9]]. These stems, available in several designs such as the Fitmore stem (Zimmer, Warsaw, IN, USA), aim to offer enhanced bone preservation by reducing stress shielding and enabling minimally invasive procedures. Not only do these short stems preserve bone and soft tissue more effectively but they also create better conditions for future revisions by allowing placement in a more flexed position to conserve the femoral neck bone stock [9]. Besides, the curved design of Fitmore stem (Zimmer, Warsaw, IN, USA) is ideal for less invasive procedures and aids in the preservation of the medial side of the great trochanter and the abductor muscles, thereby reducing operative time and enhancing postoperative rehabilitation [9,10] (Fig. 1).

The clinical and radiologic analysis concerning the survival of Fitmore stem are mostly limited to short-term to mid-term results in small quantities [9,[11], [12], [13], [14], [15], [16], [17], [18], [19], [20]]. And there is only 1 prior study on 80 hips suggesting good survival rates at 10-year follow-up; however, this study had a small patient population that might not be generalizable to a wider population [21]. Initial findings indicate promising outcomes such as reduced stress shielding, improved physiological proximal load transfer, lower micromotion, and less subsidence. Despite these encouraging initial outcomes, concerns have been raised about cortical hypertrophy (CH), reported in 29%-63% of cases, which may pose long-term issues [14,16]. Moreover, data about the long-term outcomes and survival of these stems in larger, younger populations are scarce.

In response to this gap in the current body of knowledge, our study endeavors to present a comprehensive analysis of the mid-term to long-term outcomes in our patients who underwent THA using the Fitmore stem via a direct anterior approach (DAA) by a single surgeon from 2010 to 2019 to address [1] complications and survivorship [2], the clinical and [3] radiologic outcomes, and [4] assessment of risk factors associated with stem revision.

## Material and methods

### Study design and patients

This single-center, retrospective study aimed to evaluate the mid-term to long-term outcomes of THA performed with a cementless trochanteric sparing short stem through a DAA. Our Institutional Review Board (IR.TUMS.IKHC.REC.1400.220) reviewed and approved the study protocol, and written informed consent was obtained from all participants. All surgical procedures were either conducted by an experienced orthopaedic hip surgeon or supervised by him directly (conducted by a hip surgeon fellow), ensuring consistent surgical technique throughout the study period. The inclusion criteria consisted of all patients undergoing primary THA in our hospital. Primary indications of THA are painful hip unresponsive to conservative treatments demonstrated in Table 1. The exclusion criteria consisted of patients who had undergone previous hip surgeries, patients who died during follow-up period, and patients who were not available at the latest follow-up.

**Table 1:** Demographic and preoperative data.

Demographics and indications	Mean ± SD or N (%)
Hips (patients)	755 (667)
Female:male	412 (54.6):343 (45.4)
Right:left	376 (49.8):379 (50.2)
Follow-up (y)	10.52 (range: 3-13)
Age (y)	48.9 ± 14.2 (range: 18-83)
Weight (kg)	71.5 ± 11.3
Height (cm)	166.8 ± 9.6
BMI (kg/m^2^)	25.8 ± 4.1
Primary diagnosis	
Osteoarthritis	201 (26.5)
AVN	197 (26.2)
DDH	249 (33.0)
Femoral neck fracture	32 (4.2)
Acetabular fracture	22 (2.9)
FAI	26 (3.4)
RA	14 (1.9)
SLE	6 (0.8)
Septic	4 (0.5)
Hemophilia	2 (0.3)
Hip ankylosis	2 (0.3)

AVN, avascular necrosis; BMI, body mass index; DDH, developmental dysplasia of the hip; FAI, femoral acetabular impingement; RA, rheumatoid arthritis; SD, standard deviation; SLE, systemic lupus erythematous.

**Figure 2:**
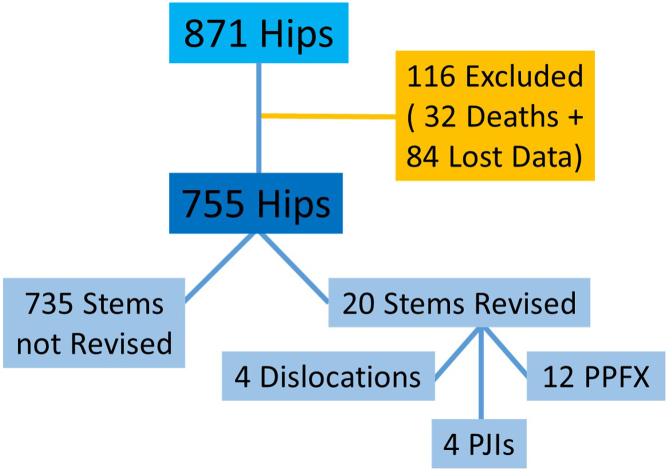
Patient follow-ups. The overall path of included patients and causes of stem revisions are demonstrated. PJI, periprosthetic joint infection; PPFX, periprosthetic fracture.

Between 2010 and 2019, a total of 853 THAs were performed using the Fitmore stem. The average follow-up period was 10.52 years (range: 3-13 years with median of 8 years). During this period, 32 patients (3.6%) passed away, and 84 patients (9.6%) were lost to follow-up. Consequently, a cohort of 755 hips (667 patients) was finally included in the study (Fig. 2). The average age at the time of the index surgery was 48.9 ± 14.2 years, with a gender distribution of 54.6% females and 45.4% males. Table 1 lists the preoperative diagnoses and demographic characteristics of the patients.

### Implant and surgery

The Fitmore is an uncemented curve stem classified as trochanteric sparing according to neck resection level, which is available in 4 neck angle options: A (140°), B (137°), extended B (129°), and C (127°), each in 14 sizes. Calcar radius and offsets are fixed in each group regardless of stem size and length. It has a triple-tapered design which helps achieving press-fit fixation at metaphyseal level (Fig. 1).

**Figure 3:**
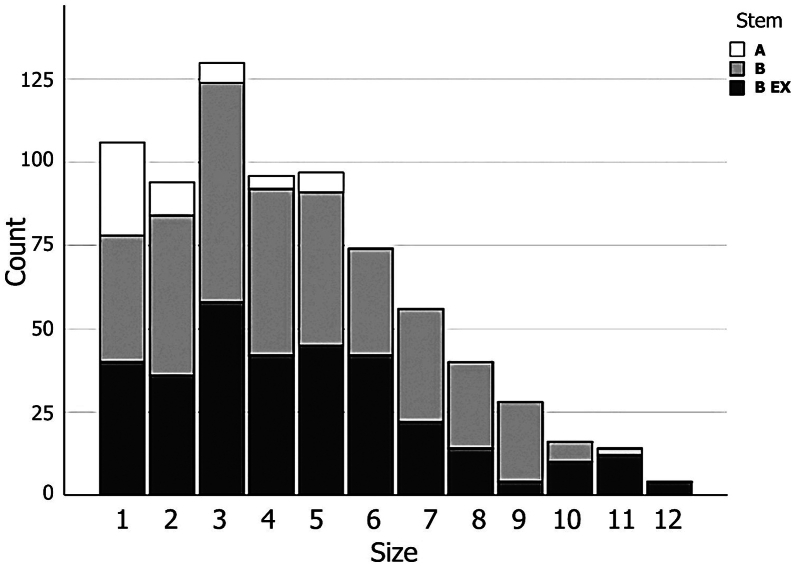
Stem type and size distributions.

Standardized preoperative planning was done for each patient using MediCAD ver3.5 (Hectec, Germany) operation planning software. Surgery was performed by a single surgeon, the senior author (S. M. J. M) with the same protocol for each case. DAA was used in all cases in supine position. After serial femoral broaching, the last stem size which had no axial and rotational motion was chosen for definite fixation. Stem type and size distributions are demonstrated in Figure 3.

The femoral stem was employed in conjunction with 2 different acetabular components: the Continuum (Zimmer Biomet, Warsaw, IN, USA) and the Trilogy (Zimmer Biomet, Warsaw, IN, USA), used in 48.6% and 51.4% of the cases, respectively. Both of these systems interfaced with a highly cross-linked polyethylene component (Zimmer Biomet, Warsaw, IN, USA) (Table 2).

**Table 2:** Implant details.

Component types	N (%)
Stem type	
A	54 (7.2)
B/B ext.	372 (49.3)/329 (43.6)
C	0
Cup type & size	
Continuum	367 (48.6)
Triology	388 (51.4)
Cup size	Median 52 (range: 46-64)
Head size	
28	4 (0.6)
32	113 (15)
34	5 (0.65)
36	529 (70.1)
40	104 (13.8)
Neck length	
3.5	56 (7.4)
0	332 (44)
3.5	345 (45.7)
7	22 (2.9)

Per our hospital’s guidelines, patients received antibiotic prophylaxis in the form of an initial dose of 1 g Cefazolin prior to the surgical procedure, followed by 2 more doses within the subsequent 24 hours. To prevent blood clotting, we adhered to a thromboprophylaxis protocol that involved administering 325 mg of aspirin twice daily over a span of 4 weeks. The surgical approach did not involve the use of any drains. Postoperatively, patients were encouraged to bear weight as their comfort permitted, using walkers for support. Provided no complications arose, they were discharged the day after surgery. Follow-up examinations were then scheduled at 2-4 weeks, and again at 3, 6, and 12 months, with further checks carried out annually or biennially.

### Clinical and radiologic assessment

Functional assessment was done at the latest visit by an independent research assistant who was not in index surgeries, documented through questionnaires of Harris Hip Score (HHS), Western Ontario and McMaster Universities Osteoarthritis Index (WOMAC), and visual analog scale (VAS) for hip pain. HHS results were classified as excellent (91-100), good (81-90), fair (71-80), and poor (< 70) and WOMAC was scaled from 0 (no complaints) to 96 (the worst).

**Figure 4:**
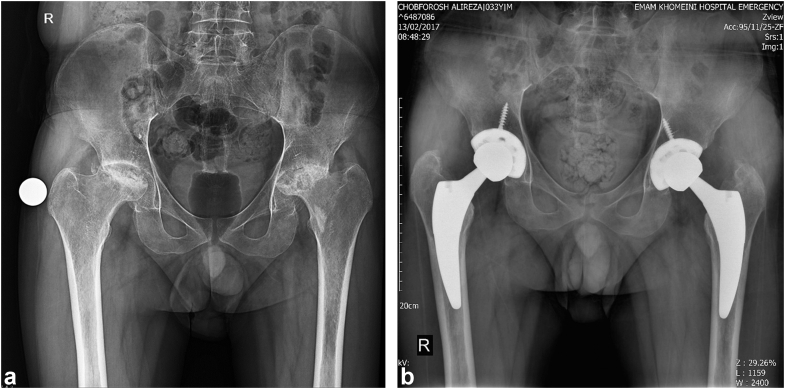
Radiographs present a case of 33-year-old patients who had bilateral avascular necrosis of femoral heads. (a) AP pelvic view shows Ficat stage IV AVN of femoral heads and Dorr type B femoral bones. (b) AP pelvic view of same patients 11 years postoperatively revealed well-fixed acetabular and femoral component. AP, anterior-posterior; AVN, avascular necrosis.

Radiographic evaluations were performed using a standardized anterior-posterior pelvic x-ray, angled at 15° of internal rotation, and a lateral hip x-ray. These images were captured preoperatively, immediately postoperatively, and at 2 years interval via the Picture Archiving and Communication System (Fig. 4). For calibration, a 25-mm radiopaque ball was placed at the level of the great trochanter between the legs during the preoperative x-rays. In subsequent images, the femoral head diameter served as the calibration reference.

Radiographic measurements were performed using the MediCAD v. 3.5 software (Hectec, Germany), as outlined in previously published methodologies [22]. Two independent observers, uninvolved in the index surgeries and blind to each other’s evaluations, were responsible for these measurements. They assessed a range of parameters including stem loosening [23], subsidence, radiolucent lines (RLLs), CH [14], osteolysis, stress shielding [24], heterotopic ossification, sagittal and coronal malalignments [25], Caput-Collum-Diaphyseal angle, femoral offset, cup inclination and anteversion angles, Dorr type of proximal femur [26], and limb length difference.

### Statistical analysis

Statistical computations were executed using SPSS software, version 23. The threshold for statistical significance was set at a *P* value of less than .05. Qualitative data were presented as frequencies and percentages, with comparisons drawn using Chi-square tests or Fisher’s exact tests, as appropriate. Quantitative data, on the other hand, were reported as means accompanied by standard deviations or as ranges. These data were compared using Student’s *t*-tests, Mann-Whitney U tests, analysis of variance, or Kruskal-Wallis tests, depending on the distribution of the data. Additionally, binary logistic regression was employed to facilitate multivariate analyses.

Prosthesis survival was evaluated using Kaplan-Meier analyses, with both all-cause stem revision and aseptic stem revision as defined end points. The analysis provided survival estimates with 95% confidence intervals (CIs) over a follow-up period averaging 10.5 years.

## Results

### Survival analysis and complications

**Figure 5:**
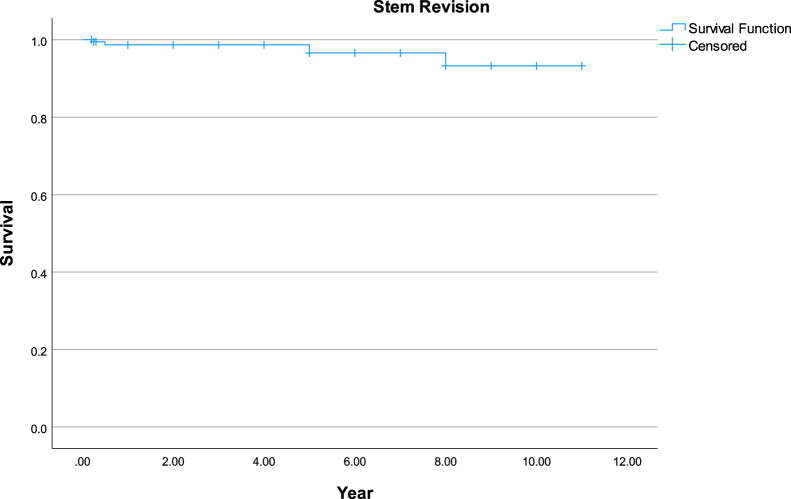
Kaplan-Meier survival analysis of total hip arthroplasty revisions using stem revision at all cause as end point. This graph shows survival rate of 92.11% (95% CI: 86.47%-97.74%) at mean follow-up of 10.51 years. CI, confidence interval.

**Figure 6:**
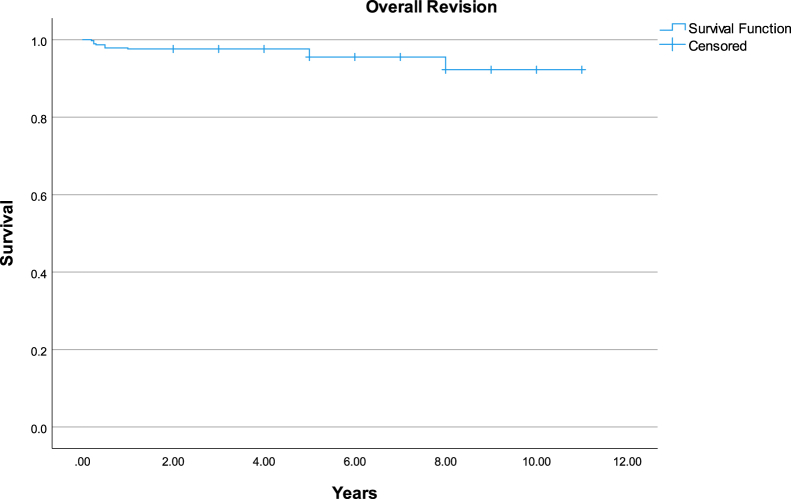
Kaplan-Meier survival analysis of total hip arthroplasty revisions using stem aseptic loosening as end point. This graph shows survival rate of 100% at mean follow-up of 10.51 years.

Using Kaplan-Meier analysis, survival rates for implants over an 11-year follow-up period were determined. Taking into account all causes of revision, the Fitmore stem survival rate was 92.11% (95% CI: 86.47%-97.74%), as shown in Figure 5. In the specific scenario of stem revision due to aseptic loosening, the Fitmore stem displayed a survival rate of 100% at this same 11-year interval. Figure 6 portrays the overall survival rate of our THA implants over the same period, which was 91.12% (95% CI: 85.50%-96.74%).

A total of 24 hips (3.2%) required revision surgery. The underlying reasons prompting these revisions were acetabular component loosening (4 hips, 0.5%) treated by acetabular component revision only, and recurrent instability (4 hips, 0.5%), periprosthetic femoral fractures (12 hips, 1.6%), and periprosthetic joint infections (PJIs) (4 hips, 0.5%) managed by stem revision only procedures. Notably, there were no revisions of the femoral component due to aseptic loosening (Table 3).

**Table 3:** Complications.

Revision causes and complications	Mean ± SD or N (%)
Revision due to	24 (3.2)
Acetabular aseptic loosening	4 (0.5)
Dislocation	4 (0.5)
PPFX	12 (1.6)
PJI	4 (0.5)
Thigh pain	13 (1.7)
PJI	6 (0.8)
HO (0/1/2/3/4)	620/93/22/20/0
Symptomatic DVT	0
Intraoperative fracture	3 (0.4)
Dislocation	7 (0.9)
Femoral nerve palsy	5 (0.7)
PPFX	18 (2.3)

PJI, periprosthetic joint infection; PPFX, periprosthetic fracture; SD, standard deviation; HO, heterotopic ossification; DVT, deep vein thrombosis.

We observed 7 instances of acute hip dislocation, 3 of which were addressed via closed reduction and 1 through open reduction. Consequently, 3 cases necessitated revision surgery. Intraoperative longitudinal fractures during stem insertion were noted in 13 patients, each managed effectively with cerclage wires without the need for further intervention. Five patients exhibited femoral nerve palsy without any instances of sciatic nerve palsy. The femoral nerve palsy cases resolved spontaneously within a 3-month period, with no lasting complications. There were no reported instances of symptomatic deep vein thrombosis. Six occurrences of PJIs were identified, with 2 patients responding to antibiotics and joint lavage treatment. Unfortunately, 4 cases necessitated 2-stage revision surgery (Table 3).

### Clinical outcome

The clinical evaluation revealed a notable improvement in the HHS, with scores rising markedly from a preoperative average of 40.3 ± 13.4 to a postoperative average of 93.1 ± 7.2 (*P* value < .001). At the most recent follow-up, the majority of patients (638 hips or 84.5%) achieved “excellent” HHS scores. A smaller number of patients (64 hips or 8.5%) had “good” scores, while 42 hips (5.6%) resulted in “fair” scores. Only a few patients (11 hips or 1.5%) had “poor” HHS scores, although it’s important to note that none of these scored less than 64. Further investigation on the cases with “poor” results revealed varied issues. One patient was dissatisfied due to PJI, which led to the complete removal of the implant following persistent infection. Another patient experienced recurring hip dislocation, necessitating revision surgery with a constrained acetabular liner. Seven cases involved a high-grade heterotopic ossification, with 6 of these patients having a previous diagnosis of developmental dysplasia of the hip (DDH), and 1 with a prior acetabular fracture, who refused surgery to remove the HO. The remaining 2 cases with poor results had a history of bilateral DDH, but their main symptoms were related to weakness and a limp due to poor abductor muscle strength. These patients underwent physical therapy and exercises to strengthen their abductor muscles.

Significant improvements were observed in the preoperative to postoperative scores for both the WOMAC and the VAS for pain. The WOMAC score exhibited a marked decrease from 58.9 ± 14.2 preoperatively to 9.6 ± 6.4 postoperatively (*P* value < .001). Concurrently, the VAS pain score exhibited a substantial reduction from 6.7 ± 1.0 in the preoperative state to 0.2 ± 0.5 postoperatively (*P* value < .001) (Table 4).

**Table 4:** Patient-reported outcomes.

Score	Preoperative	Last follow-up	*P* value
WOMAC	58.9 ± 14.2	9.6 ± 6.4	<.001
HHS	40.3 ± 13.4	93.1 ± 7.2	<.001
Excellent	0	638 (84.5)	-
Good	0	64 (8.5)	-
Fair	6 (0.8)	42 (5.6)	-
Poor	749 (99.2)	11 (1.5)	-
VAS pain	6.7 ± 1.0	0.2 ± 0.5	<.001

HHS, harris hip score; VAS, visual analog scale; WOMAC, Western ontario and McMaster universities osteoarthritis index.

### Radiologic outcome

The results of our radiographic analysis, conducted preoperatively and postoperatively, are outlined in Table 5. This analysis first employed the Dorr classification, which classified 12.2% of hips as Type A, 83.8% as Type B, and 4% as Type C.

**Table 5:** Radiological assessment.

Radiographic measurement	Mean ± SD or N (%)
Dorr type	
A	92 (12.2)
B	633 (83.8)
C	30 (4.0)
Cup inclination	43.3 ± 5.0 (range: 18-60)
Cup anteversion	15.1 ± 3.0 (range: 8-23)
FO pre (post)	31.7 ± 7.4 (39.7 ± 3.8)
LLD pre (post)	9.9 ± 6.7 (1.7 ± 1.8)
CCD angle	132.0 ± 5.6 (range: 110-151)
Coronal stem alignment	
Neutral (within ± 3)	705 (93.3)
Valgus	8 (1.1) valgus degree: 4.5 ± 0.5
Varus	42 (5.6) varus degree: 5.6 ± 1.6
Sagittal stem alignment	
Neutral (within ± 3)	599 (79.3)
Flexion	156 (20.7)
Stem subsidence (mm)	0.61 ± 0.87
Stem loosening	
Stable	749 (99.2)
Fibrous	6 (0.8)
Unstable	0
Stress shielding grade (0/1/2/3/4)	448 (59.3)/239 (31.6)/60 (8.0)/8 (0.1)
Osteolysis	0
Radiolucent line (RLL) > 2 mm	0
Radiolucent line (RLL) < 2 mm	24 (3.2)
Zone 3	10 (1.3)
Zone 4	10 (1.3)
Zone 5	14 (1.9)
Cortical hypertrophy (CH)	115 (15.2)
Zone 3	93 (12.3)
Zone 4	14 (1.9)
Zone 5	44 (5.8)
Pedestal	12 (1.6)

CCD, caput-collum-diaphyseal; LLD, limb length difference; SD, standard deviation; FO, femoral offset; RLL, radiolucent line.

Regarding stem alignment, 6.7% of stems were discovered to be malpositioned in the coronal plane, indicating more than 3° in both varus (5.6%) and valgus (1.1%). Although, these misalignments had no significant impact on clinical outcomes (*P* value: .45).

The analysis also assessed stem subsidence, with the mean measurement being 0.6 ± 0.87 and no instance surpassing 5 mm. RLL and CH were only found in Gruen zones 3 to 5, as detailed in Table 5. These conditions were significantly related to stem revisions. Conversely, the pedestal sign, although noted as positive in 1.6% of cases, did not show any significant relationship with either stem revision or clinical outcomes (*P* value: .57).

### Comparison analysis

Table 6 details the associations found between various variables and stem revisions in a sample of 755 stems with significance level of *P* value < .05. Besides, we conducted a multivariate regression analysis on the previously significant associations and found only the presence of CH to be significantly associated with stem revisions (*P* value: .04; odds ratio: 3.57; CI: 1.042-12.24).

**Table 6:** Associations of stem revision.

Variable	Nonrevised stems (735) no. (%)	Revised stems (20) no. (%)	*P* value
Sex			.16
Female	398 (96.6)	14 (3.4)	
Male	337 (98.3)	6 (1.7)	
Age	48.7 ± 14.1	54.5 ± 14.8	.07
Side			.99
Right	365 (97.1)	11 (2.9)	
Left	370 (97.6)	9 (2.4)	
BMI			.065
Underweight < 18.5	12 (100)	0	
Normal 18.5-24.9	303 (96.2)	12 (3.8)	
Overweight 25-29.9	326 (97.6)	8 (2.4)	
Obese > 30	94 (100)	0	
Stem type			.21
A	54 (100)	0	
B	364 (97.9)	8 (2.1)	
B ext.	317 (96.4)	12 (3.6)	
Bilateral			.72
Yes	172 (97.7)	4 (2.3)	
No	563 (97.3)	16 (2.7)	
Diagnosis			.008
Osteoarthritis	194 (97)	6 (3)	
AVN	194 (98)	4 (2)	
DDH	245 (98.4)	4 (1.6)	
Femoral neck fractures	28 (87.5)	4 (12.5)	*P* (post-hoc) <.001
Acetabular fracture	22 (100)	0	
FAI	24 (92.4)	2 (7.6)	
Other^a^	28 (100)	0	
Dorr			.39
A	88 (95.7)	4 (4.3)	
B	617 (97.5)	16 (2.5)	
C	30 (100)	0	
CCD	131.89 ± 5.61	134 ± 2.79	.004
Coronal stem malalignment			.54
Yes	48 (96)	2 (4)	
No	687 (97.5)	18 (2.5)	
Sagittal stem malalignment			.23
Yes	154 (98.7)	2 (1.3)	
No	581 (97)	18 (3)	
Stress shielding			.41
0	427 (97.3)	12 (2.7)	
1	214 (98.2)	4 (1.8)	
2	83 (91)	4 (9)	
3	11 (100)	0	
HO brooker			.06
0	602 (97.1)	18 (2.9)	
1	93 (100)	0	
2	22 (100)	0	
3	18 (90)	2 (10)	
Subsidence > 2 mm			.006
Yes	12 (85.7)	2 (14.3)	
No	723 (97.6)	18 (2.4)	
RLL			.002
No	713 (97.5)	18 (2.5)	
Yes	22 (91.7)	2 (8.3)	
Zone 3	10 (100)	0	.59
Zone 4	8 (80)	2 (20)	.001
Zone 5	12 (85.7)	2 (14.3)	.006
CH			<.001
No	630 (98.4)	10 (1.6)	
Yes	105 (91.3)	10 (8.7)	
Zone 3	85 (91.4)	8 (8.6)	<.001
Zone 4	12 (85.7)	2 (14.3)	.006
Zone 5	38 (86.4)	6 (13.6)	<.001
Pedestal			.57
Yes	12 (100)	0	
No	723 (97.4)	20 (2.6)	
Stem loosening			.009
Stable	731 (97.6)	18 (2.4)	
Fibrous	4 (66.7)	2 (33.3)	
Unstable	0	0	
LLD			
Preoperative	9.85 ± 6.67	11.6 ± 6.22	.24
Postoperative	1.72 ± 1.83	1.30 ± 1.59	.31

AVN, avascular necrosis; BMI, body mass index; CCD, Caput-Collum-Diaphyseal; CH, cortical hypertrophy; DDH, developmental dysplasia of the hip; FAI, femoral acetabular impingement; LLD, limb length difference; RLL, radiolucent line; HO, heterotopic ossification.

a Others: RA, SLE, septic arthritis, hemophilia, previous hip fusion.

## Discussion

The present study sought to assess the mid-term to long-term clinical and radiographic outcomes following THA performed with a cementless trochanteric sparing short stem through a DAA. The Fitmore stem demonstrated a favorable survival rate of 92.11% on an average of 10.5 years of follow-up, and patients achieved significant improvements in function and pain scores postoperatively demonstrated in Table 4. The common reasons of stem revisions were accounted as recurrent instability, periprosthetic femoral fractures, and PJIs. There was no revision required due to aseptic loosening. Radiographic outcomes also showed an acceptable rate of complications and negligible stem loosening and subsidence, underscoring the overall safety and effectiveness of this surgical technique.

The results of our comprehensive analysis of 755 THA procedures using this stem were encouraging, showing improvements in HHS, and WOMAC scores and very high survival rate of 92.11%. However, previous research on the Fitmore stem was limited to shorter follow-up periods or smaller patient groups (Table 7). Also, there is only 1 prior study on 80 hips suggesting good survival rates on 10-year follow-up which may lack generalizability to a larger population. A comprehensive comparison of the factors investigated in our study with those in other research has been provided in the supplementary material.

**Table 7:** Demographics and implant survivorship for Fitmore stem in previous studies.

Study	Level of evidence	No. of hips	No. of patients	Mean age (y)	Mean follow-up (y)	Stem survivorship
Gutske, 2012 [9]	IV	500	500	67	1.3	99.4%
Von Roth, 2014 [27]	IV	40	40	60.1	0.1	Not reported
Gasbarra, 2014 [19]	IV	33	33	62.3	1	100%
Maier, 2015 [12]	IV	100	96	59	3.3	100%
Acklin, 2016 [7]	IV	24	24	64	2	Not reported
Freitag, 2016 [28]	IV	57	57	56.8	1	Not reported
Thalmann, 2019 [16]	IV	96	95	62	5	99%
Inmann, 2019 [14]	IV	246	233	61	8.6	93.7%
Meyer, 2019 [15]	IV	140	140	53.5	5	Not reported
Meyer, 2020 [11]	IV	57	57	57.2	5	Not reported
Luger, 2021 [18]	IV	106	106	56.8	0.25	Not reported
Fujji, 2022 [13]	IV	241	241	65	3	Not reported
Ribly, 2023 [20]	IV	70	35	59	5	Not reported
Schader, 2023 [21]	IV	80	78	60.7	10	99%
Our study	IV	755	667	48.9	10.5	92.11%

Compared to prior studies (average participant age: 61 years) [9,11,12,[14], [15], [16],^27^,^28^], our research included younger patients (average age: 48.5 years), possibly due to the prevalence of DDH and avascular necrosis which together accounted for 59.2% of primary diagnoses. We observed that factors such as RLL (*P* value: .002) and CH in any zones (*P* value < .001), stem subsidence more than 2 mm (*P* value: .006), fibrous stable stems per the Engh criteria (*P* value: .09), and femoral neck fracture (*P* value < .001) were significantly associated with stem revisions as described in details in Table 6.

Stem subsidence, a critical factor in stability [23], showed no impact on clinical scores in our study, although significant stem subsidence was associated with higher revision rate. This is in line with other studies through the literature [7,9]. The existence of RLL around the stem was also correlated with higher revisions in our study. This finding contrasts with the results of a study conducted by Maier et al., wherein RLL was not found to influence clinical outcomes [12]. However, it must be acknowledged that the prevalence of RLL in the study by Maier et al. was observed to be higher, which may account for the discrepancy between the 2 studies.

Our study revealed a correlation between stem revision and the occurrence of CH (*P* value .04; odds ratio: 3.57; CI: 1.042-12.24). Previous studies by Talmann et al. and Inmann et al. also noted that the presence of CH didn't significantly affect the clinical outcomes measured by HHS or WOMAC scores [14,16].

Our study supported previous findings that short stem designs, like the Fitmore stem, are effective in preserving femoral bone [5,11,15]. We observed minimal to moderate stress shielding in 46.1%, 22.4%, and 9% of cases, respectively. None of our cases experienced severe bone loss (stress shielding grade 3 or more) or osteolysis. These results highlight the effectiveness of the short, curved Fitmore stem in preserving bone in femur, although they didn't significantly influence clinical outcomes or stem survivability.

Periprosthetic fractures, a severe THA complication, were found to be reduced with the use of short stems. Our findings aligned with previous research, reporting a low periprosthetic fracture rate [29,30].

The present study is not without limitations. Its retrospective nature, coupled with the absence of a control group for direct comparison, may potentially introduce selection bias. Furthermore, we analyzed radiographic outcomes from 2-dimensional images, which may not reflect the true 3-dimensional placement of the stem. Another limitation is the absence of bone mineral density measurement as it was not the routine protocol of our center. Furthermore, having lost data (about 10%) which is problematic in any retrospective cohort may have affected our results which is acceptable in a long-term study but may bias our findings if all of these patients failed due to aseptic loosening for instance.

## Conclusions

This study favors the Fitmore stem’s viability as a reliable option for THA especially in younger patients, as evidenced by its commendable implant survival rates, substantial functional improvements, and low incidence of radiographic complications suggesting it has an appropriate role for bone preservation and less invasive surgical approaches such as the DAA.

## Conflicts of interest

The authors declare that they have no conflict of interest.

For full disclosure statements refer to https://doi.org/10.1016/j.artd.2025.101623.

## CRediT authorship contribution statement

**Seyed Mohammad Javad Mortazavi:** Writing – review & editing, Project administration, Data curation. **Pouya Tabatabaei Irani:** Writing – review & editing, Writing – original draft, Methodology, Data curation, Conceptualization. **Mohammad Poursalehian:** Writing – review & editing, Methodology, Formal analysis. **Mahsa Mahanrad:** Investigation, Data curation. **Peyman Mirghaderi:** Supervision, Methodology, Formal analysis, Conceptualization. **Mohammadreza Razzaghof:** Writing – review & editing. **Sadegh Saberi:** Writing – review & editing, Validation, Supervision.

## Appendix

### Detailed comparison of our findings to other studies

In 2012, Gustke et al. tracked the progress of 500 patients for 1.3 years after they had THA with the Fitmore stem. They reported impressive survival rates of 99.4% when considering stem revisions. In 2019, Inmann et al. presented the longest follow-up study for this stem, monitoring 246 hips over an average of 8.3 years. They found a survival rate of 93.7% [1,2]. In our research, we provide a more extended analysis of 755 THA procedures using the Fitmore stem. Our findings showed encouraging long-term outcomes with an average follow-up of 10.51 years (ranging from 3 to 13 years). We observed improvements in the HHS and the WOMAC from 40.3 ± 13.4 to 93.1 ± 7.2 and 58.9 ± 14.2 to 9.6 ± 6.4, respectively (with *P* < .001). The survival rate for stem revision for all reasons and aseptic stem loosening were 92.11% (95% CI: 86.47%-97.74%) and 100%, respectively.

Stem migration, also known as stem subsidence, is a crucial factor in stem stability, as outlined by Engh et al. [9]. In our study, stem subsidence was measured to be 0.61 ± 0.87 mm, and it was found to have no impact on the final HHS, WOMAC, or VAS pain clinical scores. We didn't observe any cases of progressive subsidence. However, in a study by Gutske et al. [1], it was noted that primary subsidence exceeding 2 mm occurred in 34% of cases, but never more than 8.7 mm. Following this initial subsidence, stable fixation was achieved, and no further progressive subsidence was observed. Importantly, this primary subsidence didn't affect the HHS clinical evaluation. In a similar vein, Achlin et al. conducted a radio-stereometric analysis on 34 Fitmore stem cases after 2 years and found an average subsidence of only 0.34 mm. This subsidence didn't progress after the first year and had no impact on the clinical outcomes [10]. Conversely, while stem subsidence had no impact on clinical scores in our study, we found that stem subsidence greater than 2 mm was significantly associated with stem revisions. This condition occurred in 10% of revised stem cases compared to just 1.6% in nonrevised cases.

The existence of an RLL around the stem can potentially indicate stem instability and a lack of endosteal contact between the stem and the host bone [11]. In our study, we did not observe RLLs exceeding 2 mm, but we did find RLLs less than 2 mm in 3% of our cases, located exclusively in Gruen zones 3, 4, or 5. In a study by Kutzner et al., they also identified 2.9% of cases with RLLs having a maximum width of 2 mm [12]. However, these were observed 5 times in Gruen zone 1 and once in each of zones 3, 4, 5, and 7. Similarly, Maier et al. found no RLLs greater than 2 mm around the stem in their study of 97 patients, although they observed RLLs less than 2 mm in as many as 25% of cases, particularly in Gruen zones 1, 5, and 7 [4]. In Maier’s study, these findings didn't impact clinical outcomes, which contrasts with our findings. In our study, the presence of RLLs around the stem significantly correlated with stem revisions. Specifically, 10% of femurs with RLLs ultimately required stem revision, compared to only 3% of cases without RLLs.

Our study revealed a noteworthy correlation between stem revision and the occurrence of CH. We found that 15.2% of our cases exclusively exhibited CH in specific Gruen zones, namely zone 3 (12.3%), zone 4 (1.9%), and zone 5 (5.8%). Previous studies by Thalmann et al. and Inmann et al. reported higher rates of CH, with 77% and 56% of their cases occurring in Gruen zones 3 and 5, respectively. However, like our findings, they noted that the presence of CH didn't significantly affect the clinical outcomes measured by HHS or WOMAC scores [2,6]. Both the cited studies found no significant influence of patient characteristics, prosthesis factors, or surgical approach on the final outcomes. Interestingly though, we found that using longer stems tended to result in more CH. Also, we noted that patients with CH had a significantly higher average BMI (26.8 kg/m^2^) compared to those without CH (25.5 kg/m^2^).

The creation of short stem designs was mainly driven by the goal of preserving the femoral bone [13]. Maier et al. published 2 studies in 2019 and 2020 examining bone density after THA and the protective effect of different stem designs against stress. In the first study, they assessed the bone density in the upper thigh of 140 patients, 5 years after they received either a Fitmore or a CLS Spotorno stem. They found that patients with the shorter, bone-conserving Fitmore stem experienced less bone loss than those with conventional straight stems, particularly in the areas known as Gruen zones 3 and 5 [7]. The second study looked at the gender-specific effects of the Fitmore stem on bone conservation, 5 years after THA in 57 patients [8]. They found a reduction in bone density around the entire prosthetic area, which, however, did not significantly affect the patients’ outcomes as measured by HHS and WOMAC. Interestingly, they observed more bone loss in men. This could be because of differences in how weight is distributed between men and women in this part of the thigh. However, no survival analysis was reported in their studies [7,8]. Our own research produced similar findings. We observed minimal to moderate stress shielding in 46.1%, 22.4%, and 9% of cases, respectively. None of our cases experienced severe bone loss (stress shielding grade 3 or more) or osteolysis. These results highlight the effectiveness of the short, curved Fitmore stem in preserving bone in femur, although they didn't significantly influence clinical outcomes or stem survivability.

Periprosthetic fractures (PFF), a significant complication of THA, can necessitate complicated surgeries and readmissions. The advent of short stems has reduced the risk for PFFs. Several studies have reported a significant decrease in PFFs with the use of these short stems [14,15]. Dietrich et al. observed a PFF rate of 1.6% for short stems, compared to a 6.8% rate for conventional stems, when used through a DAA [14]. Luger et al. reported a similar 1.7% rate through an anterolateral approach [15]. Our research aligns with these findings; we report a 2.3% PFF rate for short stems used through a DAA. Of the cases experiencing a PFF, 66.6% (12 of 18) required a stem revision. Among these, 5 were treated conservatively and achieved spontaneous union, necessitating no further intervention. However, 1 case required a side plate for fixation.

Our study also focused on assessing the sagittal alignment of the stem in addition to the standard coronal alignment measurements. However, no significant association was found between these factors and clinical outcomes or stem survival and supported the outcome of Yoshitani et al.’s study. They had also found no significant impact of sagittal malalignment of the stem on THA clinical or radiologic outcomes after 4.7 years of follow-up [16].

In our study, we observed a significant gender difference in the placement of stems during THA, with men more likely to have stems in flexion than women. This tendency could be attributed to the challenges of femoral exposure, potentially exacerbated by a significantly higher average weight in men (74.2 kg) compared to women (69.25 kg). Although this placement of the stem in flexion did not affect the outcomes in our research, we underscore the importance of maintaining proper stem alignment in THA. This alignment is crucial for preventing potential complications such as dislocation and impingement. Further supporting the importance of precise alignment, a study by Muller et al. found that each 5° increment of stem flexion corresponded to a 10° increase in the functional anteversion of the stem [17]. This correlation could theoretically impact dislocation and impingement rates, emphasizing the critical role of accurate stem placement in THA.

## Appendix A Supplementary data

Conflict of Interest Statement for Mahanrad

Conflict of Interest Statement for Mirghaderi

Conflict of Interest Statement for Mortazavi

Conflict of Interest Statement for Poursalehian

Conflict of Interest Statement for Razzaghof

Conflict of Interest Statement for Saberi

Conflict of Interest Statement for Irani
